# Supplementary material for: Proteomics and C9orf72 neuropathology identify ribosomes as poly-GR/PR interactors driving toxicity
Source: Life Sci Alliance. 2018 May 16;1(2):e201800070. doi: 10.26508/lsa.201800070 (PMC6238541; doi:10.26508/lsa.201800070)
Supplement: Supplementary file 4 [file LSA-2018-00070_SdataF1.pdf]

SOURCE DATA for Fig. 4B

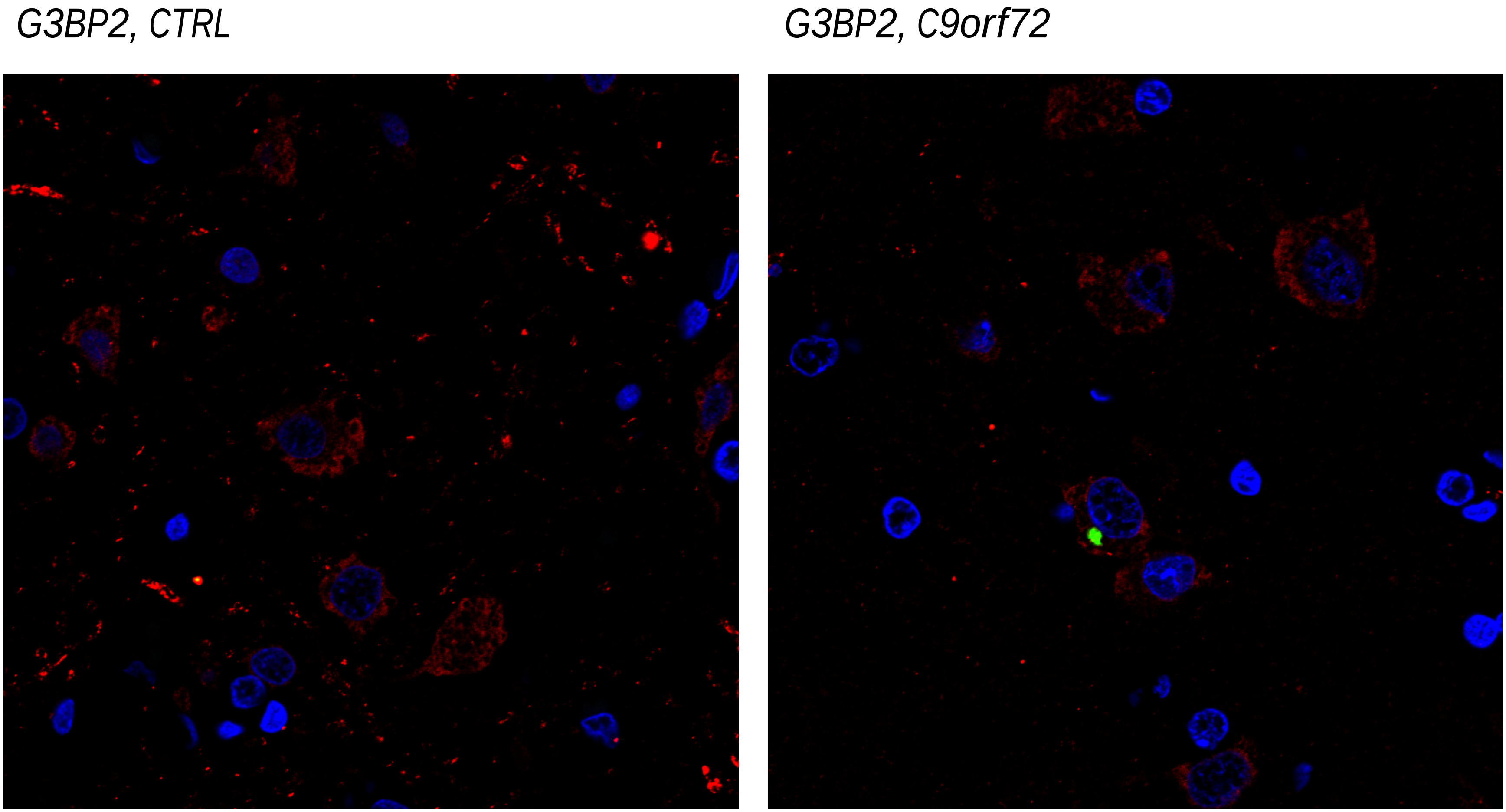

G3BP2, DAPI, GR

SOURCE DATA for Fig. 4B

TIAR, CTRL

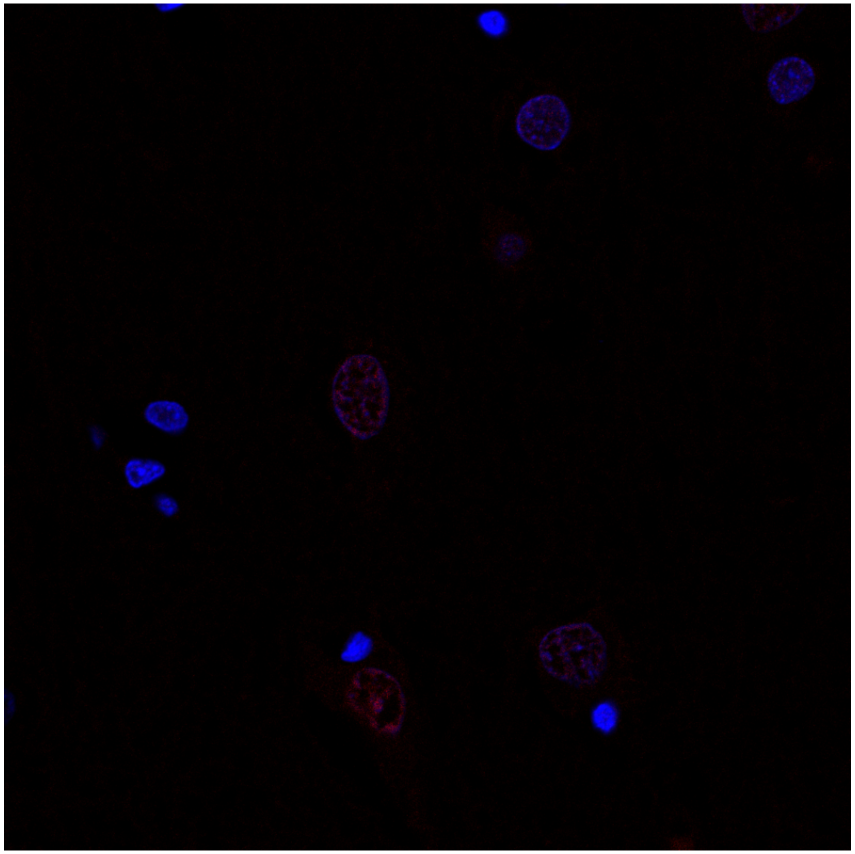

TIAR, C9orf72

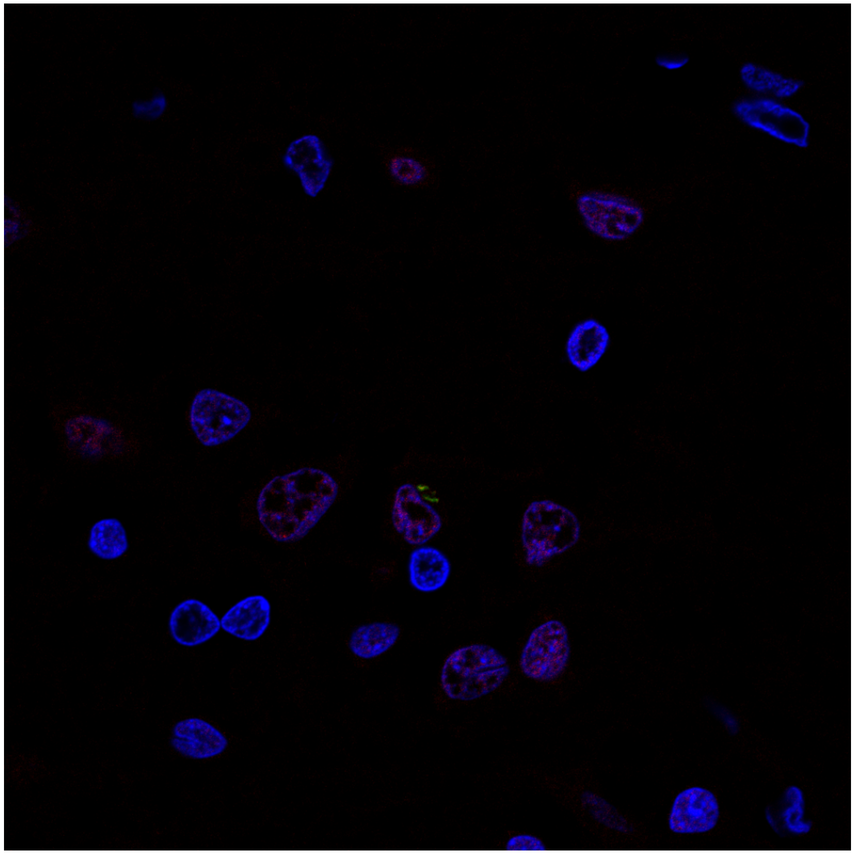

TIAR, DAPI, GR

SOURCE DATA for Fig. 4B

YBX1, CTRL

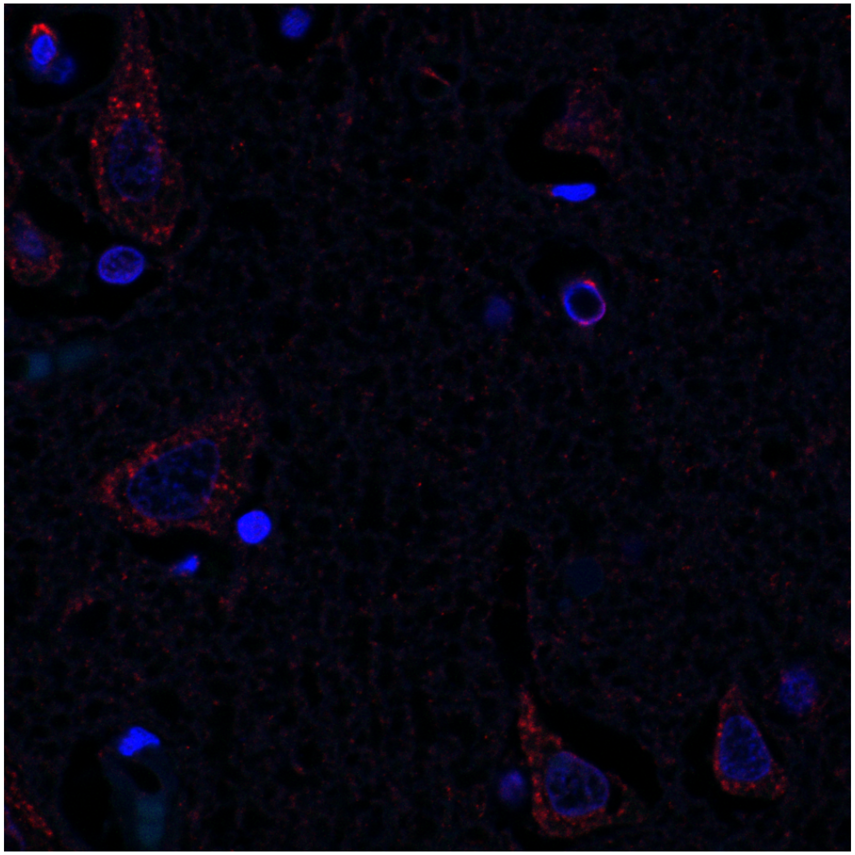

YBX1, C9orf72

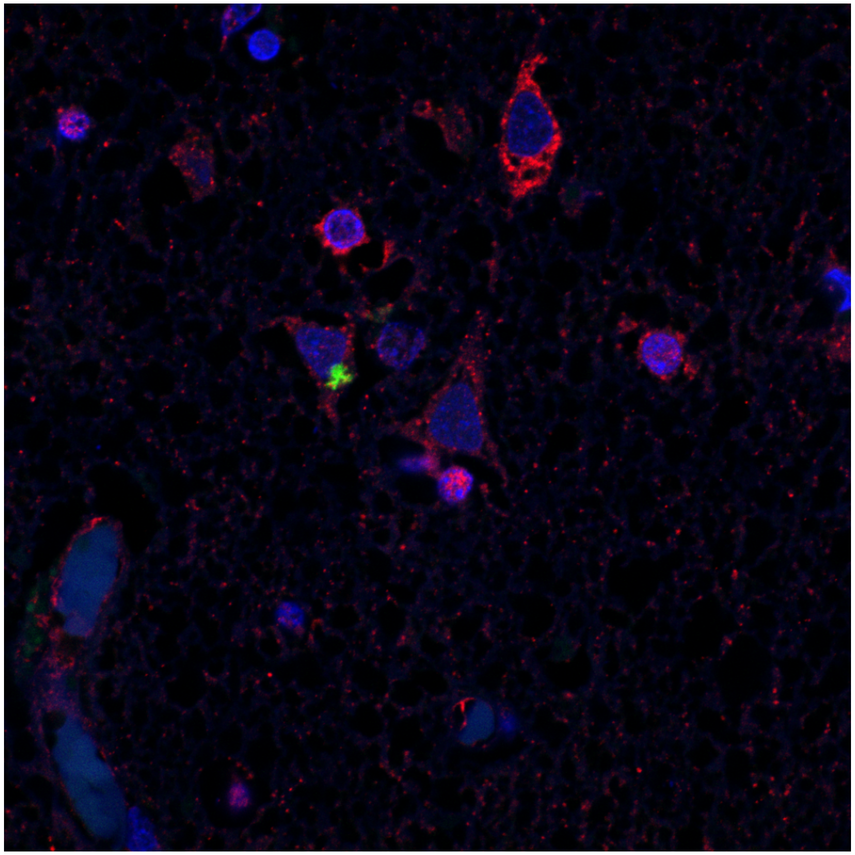

YBX1, DAPI, GR

SOURCE DATA for Fig. 4B

STAU2, CTRL *STAU2, C9orf72*

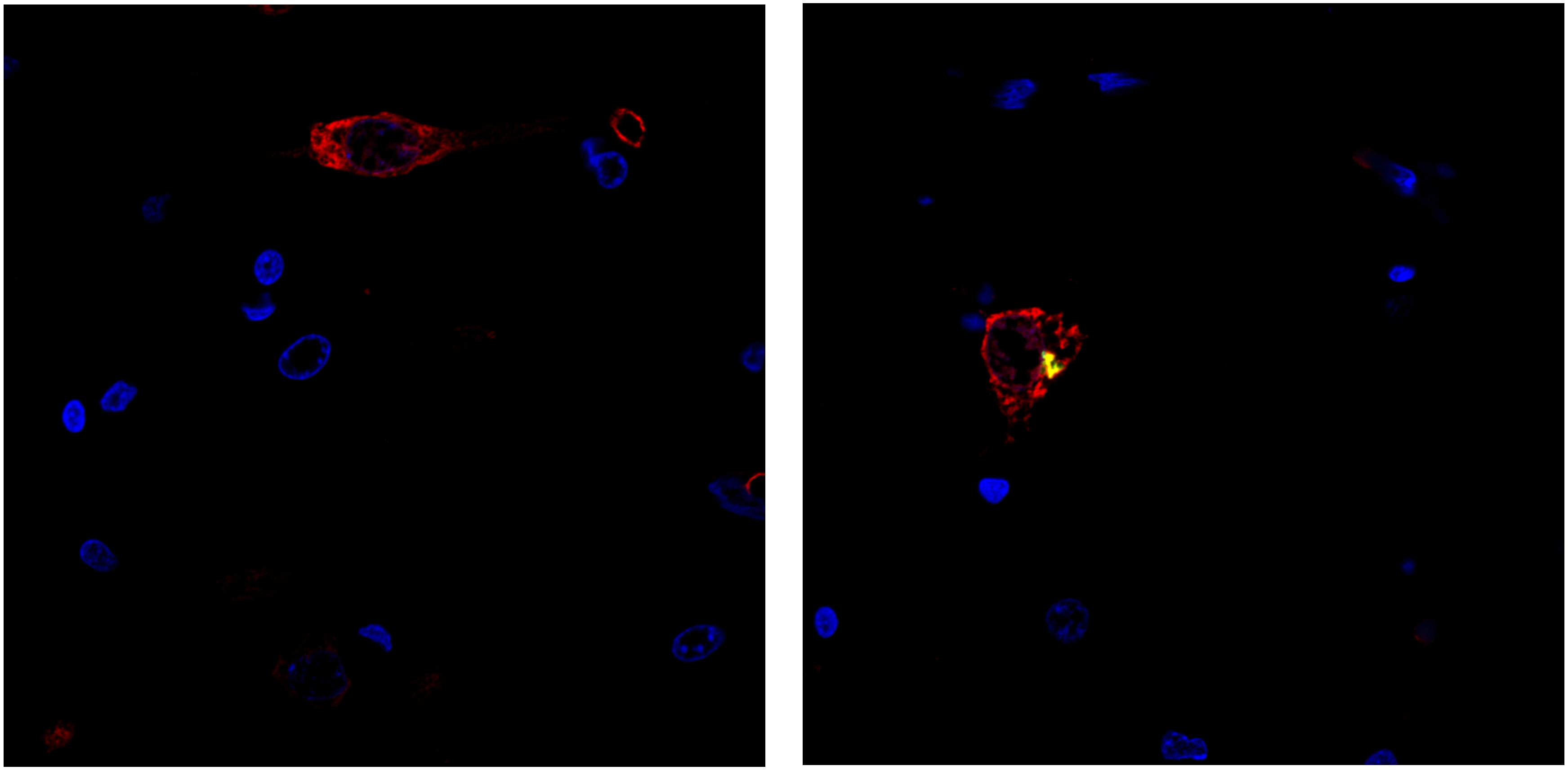

STAU2, DAPI, GR
